# Mobile Technology to Increase HIV/HCV Testing and Overdose Prevention/Response among People Who Inject Drugs

**DOI:** 10.3389/fpubh.2017.00217

**Published:** 2017-08-23

**Authors:** Ian David Aronson, Alexander Bennett, Lisa A. Marsch, Theodore C. Bania

**Affiliations:** ^1^National Development and Research Institutes, New York, NY, United States; ^2^Digital Health Empowerment, Brooklyn, NY, United States; ^3^Center for Technology and Behavioral Health, Geisel School of Medicine at Dartmouth, Lebanon, NH, United States; ^4^Icahn School of Medicine at Mount Sinai, Mount Sinai St. Luke’s, New York, NY, United States

**Keywords:** HIV, HCV, overdose, multimedia, tablet computers, video education, outreach

## Abstract

The United States faces dramatically increasing rates of opioid overdose deaths, as well as persistent ongoing problems of undiagnosed HIV and HCV infection. These problems commonly occur together in substance using populations that have limited, if any, access to primary care and other routine health services. To collectively address all three issues, we developed the Mobile Intervention Kit (MIK), a tablet computer-based intervention designed to provide overdose prevention and response training and to facilitate HIV/HCV testing in community settings. Intervention content was produced in collaboration with experienced street outreach workers who appear onscreen in a series of educational videos. A preliminary pilot test of the MIK in a Bronx, NY street outreach syringe exchange program found the MIK is feasible and highly acceptable to a population of people who inject drugs. Participants accepted HIV and HCV testing post-intervention, as well as naloxone training to reverse overdose events. Pre-post tests also showed significant increases in knowledge of overdose prevention, HIV testing procedures, and asymptomatic HCV infection. Future iterations of the MIK can be optimized for use in community as well as clinical settings nationwide, and perhaps globally, with a focus on underserved urban populations.

## Introduction

Drug overdose deaths in the United States nearly tripled between 1999 and 2014 (1). During 2015 more than 50,000 people died from overdose nationwide, and almost two-thirds (63.1%) involved opioids (1). Research with opioid-using populations has established that social and structural factors, including poverty, homelessness, or periods of abstinence from opioids due to incarceration, hospitalization, or inpatient drug treatment, can increase vulnerability for overdose (2–7). Moreover, interpersonal relationships and events, social supports, and life turning points (8–10) can influence substance use practices and behaviors in ways that can contribute to an overdose event (11). Additionally, the increased presence of fentanyl (12, 13) possible in any given sample of heroin (14, 15) has contributed greatly to overdose risk.

Many people who inject drugs (PWID) may lack basic information about overdose prevention and response, and these knowledge deficits may contribute to overdose risk. For example, PWID may not understand that a person’s overdose risk can increase under certain circumstances due to predictable changes in tolerance (i.e., people are more likely to overdose shortly after being released from jail), or that Good Samaritan laws protect people who call 911 to report an overdose. Some people may also not know that substances they are using (e.g., Percocet) are opioids that contribute to overdose risk, especially if used with alcohol (16).

Facing reduced access to prescription opioids (POs) from medical sources, some users have turned to diverted POs. Some PO users have also transitioned to heroin use/injection, because compared to POs, heroin may be easier to obtain and less expensive (17–20). The use of heroin, especially when injected, can present additional health consequences, including overdose and HIV/HCV transmission.

In addition to overdose risks described above, PWID who take steps to prevent HIV infection (e.g., not sharing syringes; going to a syringe exchange to obtain new ones) may remain unaware that HCV can be spread by sharing other injection equipment (i.e., cookers, water, or cottons) or that people often live with HCV for years without showing symptoms. Moreover, PWID at risk for HIV may not know that HIV tests can be performed in as little as 20 min without drawing blood. In addition, even when people do understand their risk, many decline HIV and HCV testing when offered, for a range of reasons, including fear of stigma and/or mistreatment (21).

Community-based syringe exchange programs (SEPs) often provide overdose prevention/response training along with HIV/HCV education and testing in street outreach settings. SEPs are generally located in areas with high levels of injection drug use, and offer PWID opportunities to safely dispose of used syringes and obtain new ones free of charge. Many SEPs are staffed by former opioid users and also provide a range of basic health services (including HIV/HCV testing and overdose prevention) to people who otherwise may not receive care. However, even the most skilled outreach workers can only interact with a limited number of people per day, and can only be in so many places at once. As a result, even when well-trained outreach teams are present, PWID who come to an SEP to receive new syringes may not make use of other available services from which they could clearly benefit.

### Technology-Based Interventions

Our team has previously used technology-based interventions to facilitate HIV testing and related education among emergency department (ED) patients in high volume settings (22–25). EDs and community-based SEPs face a number of similar barriers to patient education and HIV testing, including limitations on staff availability in high volume settings and the challenge of working with high-risk populations who lack access to primary care. To address these issues in an ED setting, we created brief (<10 min) interventions that integrated pre-post knowledge testing with short educational videos on the importance and ease of testing for HIV. At the end of the interventions, computers asked each participant if they would like an HIV test. The first intervention targeted all ED patients (22), subsequent interventions were optimized for participants who initially declined testing (23, Aronson et al., submitted^1^), and for youth who declined testing at triage (24). In each of the above studies, we were able to increase HIV test rates by roughly 30% (22–24, see text footnote 1).

Employing pre-post knowledge testing designs helped us gain a better understanding of what participants knew and did not know at baseline (25), and how learning specific details from a video could potentially influence decisions to test (23). In a 2012 study, we recruited 160 ED patients who had all declined HIV testing at triage, and approximately one-third accepted testing after watching a video (23). Qualitative interviews with a sub-sample of patients who completed the intervention indicate participants tested after learning HIV tests could be administered without drawing blood and results could be available in 20 min (23). Interviews also indicated that participants were more comfortable accepting an HIV test offered by computer compared to an HIV test offered by a person, because they did not expect a computer to make judgments about them but thought that a person would (23).

While discussing future directions for our research, we hypothesized that a similar approach could be used to address issues of overdose prevention and response, as well as undiagnosed HIV and HCV, in community settings. We then developed the Mobile Intervention Kit (MIK), a tablet-based intervention with three separate modules (HIV, HCV, overdose prevention) that can be shown to participants individually or in any combination, to tailor content based on the needs of specific patients or the capacity of a facility. Our team had demonstrated in previous projects that brief interventions were both feasible and acceptable to patients in high volume clinical settings. We, therefore, hypothesized that a similar intervention could be successfully designed for PWID in community settings.

All three MIK modules follow the same format. Participants complete an automated knowledge pretest, watch a short educational video (<2 min) on the tablet, and then complete an automated knowledge posttest. Video content was developed in a collaborative process with SEP community health outreach workers and was guided by the Information, Motivation, Behavioral skills model (IMB).

IMB, which is frequently used in HIV prevention contexts (26, 27), posits that information alone will not precipitate behavior change, instead intervention content must also motivate recipients to achieve specific behavioral outcomes. IMB also recommends tailoring content to specific populations and risk behaviors (28). Accordingly, our team worked with SEP staff to develop short video messages that would not only educate PWID about HIV/HCV testing and overdose prevention/response but would also motivate participants to accept testing or training offered at the end of the intervention.

As soon as the videos end, the MIK presents a posttest to each participant. The HIV knowledge test was based on knowledge tests previously used by our team. The HCV knowledge test was based on the brief hepatitis C knowledge scale by Balfour et al. (29), and the overdose prevention and response knowledge test was based on recommended training guidelines and protocols developed by the New York Department of Health and Mental Hygiene.

After completing the knowledge posttests, the MIK offers each participant an HIV or HCV test, depending on the module they were enrolled in, or asks participants if they would like to receive training in how to administer naloxone and then receive a naloxone kit to take home.

Once participants respond to the offer of an HIV/HCV test or naloxone, the MIK presents a set of acceptance items, which are described further in the Section “Materials and Methods.”

### Research Questions

Two obvious sets of questions emerged during the MIK’s design and development. First, would SEP participants agree to participate in a preliminary pilot study, and if so, would they complete the intervention? Unlike an ED setting, where the mean wait time to see a physician is 1 h (more in urban and high volume environments) (30) and patients have a fair amount of downtime to participate in research, people generally come to an SEP for much shorter amounts of time. Second, would people at an SEP think the intervention was worth their time and attention? This question takes on particular importance because street outreach sites can present especially challenging environments for intervention delivery. Not only would the MIK need to be brief enough to complete during a short SEP visit, it would have to be self-contained and flexible enough to complete in an outdoor setting, private enough that participants would feel comfortable, and perhaps most challenging of all, interesting enough to warrant participants’ attention despite all the potential distractions around them.

To examine these questions, we designed an early stage pilot study to examine the MIK’s feasibility and acceptability at a working SEP site. In addition, we also included secondary measures to examine pre-post knowledge change, and programmed the tablets to offer HIV/HCV testing or naloxone training to each participant.

Thus, the goal of the current study was to examine the following primary research questions:

1. Would the MIK be feasible in a street outreach setting, and would participants find the intervention acceptable? Would PWID at a working SEP agree to participate, and if so, would they complete the intervention? If participants did complete the intervention, would the total time spent on the intervention be short enough to deliver without disrupting SEP workflows? Last, would participants who completed the intervention describe it as worthwhile?

In addition we sought to examine:

2. Participant knowledge. What did participants know at baseline, and did participant knowledge increase after watching a short video?
3. Preliminary efficacy. Did participants accept HIV/HCV testing or naloxone training offered by the tablets at the end of the intervention?

## Materials and Methods

In December 2016 outreach staff recruited participants at a Bronx, New York street outreach SEP run by New York Harm Reduction Educators.

### Sample

Outreach staff recruited a convenience sample of 31 SEP clients. The sample was 52% male, with a mean age of 47.84 years, SD = 12.12. Sixty-five percent of participants identified as White, including 55% White Latino (*n* = 17), and 10% White non-Latino (*n* = 3). Twenty-nine percent identified as Black or African-American, including 6% Black Latino (*n* = 2), and 23% Black non-Latino (*n* = 7). One participant identified as non-Latino American Indian/Alaska Native, and one participant identified as Latino Hawaiian or Other Pacific Islander.

### Recruitment

Participants were eligible to participate in the study if they were clients of the SEP aged 18 and older. Exclusion criteria were positive HIV or HCV status, as well as HIV or HCV testing within the past 2 months.

Participants were recruited during service provision hours. Data collection shifts were scheduled to coincide with service provision. SEP clients generally gathered at the site shortly before SEP staff began offering services each day. At the start of each data collection shift SEP staff explained our research to clients who were waiting for services and asked if any would be interested in participating. Staff then made a list of eligible clients who were willing to participate.

All participants provided written informed consent. Consent forms, study instruments, and procedures were approved by our research team’s Institutional Review Board. Participants were not offered incentives to test for HIV/HCV or to receive naloxone training. As compensation for their time, participants who completed the intervention received an $11 Metrocard, which would cover the cost of four trips on New York City public transit.

### Study Procedures and Materials

All data collection instruments were administered *via* tablet–computer. After providing written consent, participants were handed tablet computers with a web-based version of the MIK pre-installed, and a pair of headphones. The MIK first collected basic demographic data, including age, race/ethnicity, and gender. The MIK then presented all participants with a behavioral risk screening, separately examining substance use and sexual risk.

Once participants had responded to the demographic and risk screening items, the MIK presented one module to each participant addressing overdose prevention, HIV testing and prevention, or HCV testing and prevention. The intervention design enables program staff to select a single module for each participant, or multiple modules in any combination. As described above, the MIK administered a knowledge pretest to each participant, and then presented a short video followed by a knowledge posttest. After the posttests, the MIK asked each participant if they would, depending on the module, like an HIV test, an HCV test, or if they would like more information on naloxone. Last, the MIK presented an automated set of acceptance items. We have successfully used similar sets of acceptance items on previous projects [e.g., Ref. (23, 24)]. The items present separate scales measuring how interesting, useful, and easy to use participants find the information presented *via* the tablet-based intervention. Additional items measure how much new information the participants learn, along with how much they like the MIK, how much they understood, and how threatening they found it. The MIK presents each of the above items as clickable 10-point scales with the lowest possible value on the left, and the highest possible value on the right. For example, the scale asking participants how interesting they found the intervention is labeled “Not Interesting” on the left and “Very Interesting” on the right. Together, these scales can provide a more thorough understanding of participants’ experience.

When participants had completed the acceptance items, SEP staff collected the tablets and headphones. If participants declined naloxone training or HIV/HCV testing, their participation in the study was complete. Patients who accepted naloxone training met with an SEP staffer to learn how to administer naloxone to reverse an overdose. Participants who accepted HIV or HCV testing were privately tested inside a van at the outreach site and received their test results before leaving. All participants who tested HIV or HCV positive were referred to care by SEP staff.

## Results

### Primary Outcomes

#### Would PWID at a Working SEP Agree to Participate, and If So, Would They Complete the Intervention?

Data were collected at the SEP 1 day a week for a total of 3 weeks. Our recruitment goal was 10 people per day, and during each day of data collection far more than 10 people volunteered to take part. As noted above, SEP staff made a list of volunteers and then selected the first 10 eligible participants for the HCV and overdose prevention modules (if we had more Metrocards and more time we likely could have recruited many more). Staff recruited 11 participants to complete the HIV module. Of 31 total participants, all completed the intervention, and all but one completed all the post-intervention acceptability measures.

#### If Participants Did Complete the Intervention, Would the Total Time Spent on the Intervention Be Short Enough to Deliver without Disrupting SEP Workflows?

The mean time to completion, including watching a video and responding to all pre- and post-intervention items, was 26 min and 43 s (SD = 17 min 57 s). Two participant times were recorded as substantially longer than 1 h, possibly because staff did not immediately log the participants out of their sessions. If these two participant times are not included, the mean time to completion drops to 22 min and 57 s (SD = 10 min and 47 s).

#### Would Participants Find the Intervention Acceptable?

As described earlier, participants completed seven clickable scales measuring their response to the intervention. Responses were analyzed separately for each module. Other than responses to the question of how “threatening” participants found the intervention (lower scores meant less threatening) none of the acceptability items received a score lower than 6.8 out of 10. Although there were no significant differences in acceptability scores by content module, considerable difference emerged in responses to the question of how threatening participants found the intervention. Participants who completed the HCV module reported the intervention was far more threatening compared to participants who completed the HIV or overdose prevention modules. Please see Table 1 for more details.

**Table 1:** Participants found the HCV module more useful and more threatening compared to other intervention modules.

Acceptability scores, by module (possible range 0–10)							
	How *interesting* was the program you just completed? (0 = not interesting, 10 = very interesting)	How *useful* was the program you just completed? (0 = not useful, 10 = very useful)	How much *new information* did you learn as a result of the program you just completed? (0 = none, 10 = a great deal)	How *easy to use* was the section of the program you just completed? (0 = very difficult, 10 = very easy)	How much *did you understand* the program you just completed? (0 = not at all, 10 = very much)	How much *did you like* the program you just completed? (0 = not at all, 10 = very much)	How threatening did you find the program you just completed? (0 = not at all threatening, 10 = very threatening)
HIV module (*n* = 11)	M = 6.91; SD = 3.08	M = 8.27; SD = 2.65	M = 7.64; SD = 2.42	M = 6.82; SD = 3.19	M = 8.09; SD = 2.02	M = 7.82; SD = 3.12	M = 5.60; SD = 4.08
HCV module (*n* = 10)	M = 8.60; SD = 1.17	M = 9.00; SD = 0.94	M = 8.60; SD = 2.72	M = 8.90; SD = 1.29	M = 8.90; SD = 2.47	M = 8.30; SD = 2.75	M = 6.90; SD = 3.76
Overdose module (*n* = 10) (unless otherwise noted)	M = 8.80; SD = 1.81	M = 8.00; SD = 3.20	M = 7.90; SD = 3.11	M = 7.00; SD = 3.30	M = 8.10; SD = 3.03	M = 8.2; SD = 2.94	M = 4.78; SD = 3.77 (*n* = 9)

### Secondary Outcomes

#### What Did Participants Know at Baseline, and Did Participant Knowledge Increase after Watching a Short Video?

Pretest knowledge scores were generally high across all three modules, however, some notable baseline knowledge deficits emerged. Only half the participants enrolled in the overdose prevention module correctly answered that using heroin shortly after release from prison can increase overdose risk, because a person’s tolerance will be lower than it was before they were incarcerated. Only 6 out of the 10 participants enrolled in the HCV module correctly answered that people can live with hepatitis C for many years without knowing that they have been infected with the virus, and only 6 out of 10 correctly answered that a person can get reinfected with hepatitis C after the virus has been completely treated and cleared.

Pre-post knowledge scores increased for 12 out of 14 items. Knowledge increased significantly on two items, did not change for one, and decreased for another. Please see Table 2 for more detail.

**Table 2:** Pre-post knowledge scores increased for 12 out of 14 items.

Question	Pretest percentage correct	Posttest percentage correct	Change
**HIV knowledge test (*n* = 11)**			

Using vaseline or baby oil with condoms lowers the chance of getting HIV	72.73	63.64	*p* = 0.676
If you have sex without a condom, you’re at risk for HIV—you don’t have to inject drugs or be gay to get HIV	81.82	90.91	*p* = 0.588
HIV test results can be available in 20 min	63.64	100	*p* = 0.038*
HIV testing can be done without drawing blood	72.73	100	*p* = 0.082

**HCV knowledge test (*n*=10)**			

Studies show that more than 60% of people who inject street drugs are infected with hepatitis C	90	100	*p* = 0.343
People can live with hepatitis C for many years without knowing that they have been infected with the virus	60	100	*p* = 0.037*
Using “new” (i.e., never used before) needles, syringes, and equipment reduces the risk of being infected with hepatitis C	90	100	*p* = 0.343
Hepatitis C can be given to someone during sexual intercourse	90	100	*p* = 0.343
Once someone’s hepatitis C virus has been completely treated and cleared, that person cannot get reinfected with hepatitis C	60	90	*p* = 0.193

**Overdose prevention knowledge test (*n* = 10)**			

Using heroin with other substances, such as alcohol or sleeping pills can increase the risk of a heroin (opioid) overdose	80	80	*p* = 1
Using heroin again soon after release from prison can increase the risk of a heroin (opioid) overdose	50	90	*p* = 0.037*
Slow or shallow breathing might indicate an opioid overdose	90	90	*p* = 1
When managing an opioid overdose a person should call an ambulance	80	100	*p* = 0.168
When managing an opioid overdose a person should give naloxone (opioid overdose antidote)	70	90	*p* = 0.343

#### Did Participants Accept HIV/HCV Testing or Overdose Prevention/Naloxone Training Offered by the Tablets at the End of the Intervention?

All ten participants enrolled in the HCV module accepted HCV tests offered by the tablets at the end of the video. Two tests were reactive. Ten out of 11 participants in the HIV module (91%) accepted HIV tests offered by the tablet. One HIV test was reactive. Five out of 10 participants in the overdose prevention module (50%) agreed to receive additional training in naloxone when offered by the tablets. An additional participant told SEP staff she wanted to receive naloxone training after completing the intervention, bringing the total of participants who received naloxone training to 6 out of 10 (60%).

## Discussion

Results from our early stage pilot indicate the MIK is both feasible and acceptable in a street outreach setting. The findings that all participants completed the intervention, and that the mean time to completion was roughly 23 min, are especially encouraging. All participants had the ability to end their participation at any time, yet none chose to do so. The 23 min completion time suggests the MIK can potentially be integrated into a variety of community settings, just as our prior interventions have been shown feasible in high volume clinical environments. Some components of the MIK that required longer amounts of time (e.g., the risk screening) can potentially be streamlined in future iterations. Subsequent versions of the MIK can also enable program staff to select which components of the risk screening (i.e., substance use, sexual risk history) are administered or skipped, just as the current version enables staff to select which content modules are delivered to each participant.

Acceptability scores are also encouraging, as none of the items elicited a strong negative response. The difference, by module, in response to the question about how threatening participants found the intervention (the HCV module was described as more threatening compared to the other two) appears to warrant further examination. Participants may have been unnerved by video content explaining that people can live with HCV for years without showing symptoms, and that after successful treatment people can become reinfected with HCV. As described in the Section “Results,” 60% of participants answered these questions incorrectly at pretest. Ultimately, the improvements in HCV knowledge among our sample, along with the post-intervention uptake of HCV testing (100% of participants who completed the HCV module agreed to tests offered by the MIK post-intervention) may prove highly valuable. As noted in the introduction, PWID who already take steps to protect themselves from HIV (i.e., only using new syringes and not sharing them) may be unaware that sharing other injection equipment (e.g., cookers, cotton, or water) may leave them vulnerable to HCV.

The finding that half the participants who completed the overdose prevention module did not know at pretest that mixing heroin with alcohol or sleeping pills (e.g., ambien or benzodiazepines) can increase overdose risk aligns with previous research (6, 31, 32) and warrants particular attention. Understanding how changes in tolerance can increase risk can help people protect themselves, and for that reason, the overdose prevention video clearly describes how lowered tolerance after incarceration can create dangerous situations for the most knowledgeable and experienced PWID.

Although many face-to-face interventions already exist to individually address HIV prevention and testing, HCV prevention and testing, and overdose prevention/response, the MIK is different for three important reasons: the MIK is a highly replicable tablet-based intervention, it collectively addresses multiple persistent issues that threaten the health of PWID worldwide, and the MIK is designed to be both customizable and expandable. As mentioned in the Section “Introduction,” even the most skilled outreach workers can only interact with a limited number of clients per day, and can only be in so many places at once. Because the MIK is offered as a tablet-based intervention it can potentially enable SEPs and other care providers to offer services to an increased number of people, more effectively, without hiring additional staff.

Further, our findings from the current study align with previous research indicating that people are often more comfortable addressing sensitive issues *via* computer instead of face-to-face (23). This may prove especially important for interventions addressing potentially lethal issues such as HIV, HCV, and overdose. The fact that the MIK addresses all three may also prove especially valuable in settings that serve PWID. Many PWID have limited access to health care (33), making each opportunity to provide testing and prevention education that much more valuable.

Last, the MIK can be customized depending on the needs of a specific organization or a particular client. As described above, an easy to use menu enables program staff to select content modules individually or in any combination. The flexible nature of the MIK will also enable us to add new content modules in the future, e.g., the benefits of pre-exposure prophylaxis, without the time and expense required to create new intervention software for each additional health issue. By addressing multiple issues, the MIK may offer a highly economical means (both in terms of time and cost) to deliver behavioral health interventions to populations that might otherwise remain underserved. Thus, the MIK may prove especially valuable if delivered on a national or international scale.

The mobile video format also appears particularly well suited to the current project. In addition to participants reporting the intervention was useful, easy to understand, and contained new information, informal discussion with participants indicates learning beyond what we expected. The video mentions that mixing Percocet with heroin or alcohol can lead to an overdose. After watching the video, one participant animatedly told our team that he planned to warn his friends to be careful not to overdose on Percocet, because he and his friends had been using them without understanding how dangerous they could be (he said his friends told him not to worry, it’s just Percocet, you can’t overdose).

The fact that 6 out of 10 participants agreed to receive additional naloxone training, and to receive a take-home naloxone kit, is also highly encouraging. In the video, an SEP staff member walks viewers step-by-step through the assembly and use of an intra-nasal naloxone device. This may have made participants more amenable to receiving additional training and to taking kits with them.

A clear limitation of the current pilot is that participants may not have agreed to participate had they not received an incentive. Offering an $11 incentive to each intervention recipient as routine practice would greatly limit the sustainability and potential scalability of our design. Additional research can explore the use of smaller incentives, or possibly even integrating the MIK into routine practice at an SEP without offering any incentives.

As noted in the results section, the fact that far more people volunteered to complete an MIK module than we had planned for is highly encouraging. This may speak to the success of the intervention’s non-judgmental approach, and may also highlight the high levels of trust the street outreach workers we partnered with have established with the PWID they serve. It also may speak to the active role that many PWID take in accessing care outside clinical settings and the steps people take to protect their health (e.g., going to an outdoor SEP on a cold rainy day to get new syringes) (34, 35). Additional research is warranted to examine how the MIK can be implemented beyond a small-scale pilot.

Given the increasing number of opioid overdoses in the United States [78 people die every day from overdose (36)] and the ongoing widespread problems of undiagnosed HIV and HCV among substance users, the need for increased education and testing appear clear, as does the need for an intervention to address all three issues. The MIK is designed to be completely customizable and can, therefore, be adapted to specific populations, environments, and health behaviors. Further, different versions of the MIK can potentially be developed for a range of settings that serve substance users including SEPs, opioid treatment programs, and hospital EDs.

## Ethics Statement

This study was carried out in accordance with the recommendations of the NDRI Institutional Review Board with written informed consent from all subjects. All subjects gave written informed consent in accordance with the Declaration of Helsinki. The protocol was approved by the NDRI Institutional Review Board.

## Author Contributions

IA designed the study in collaboration with LM, AB, and TB. AB provided guidance on working with PWID populations. TB provided guidance as a toxicologist and medical expert in overdose prevention. IA wrote the first draft of the manuscript, all coauthors provided feedback.

## Conflict of Interest Statement

In keeping with the NIH STTR funding mechanism, IA is a Principal Investigator at National Development and Research Institutes and is a cofounder and part-owner of Digital Health Empowerment. Digital Health Empowerment is the grant recipient and the developer of the Mobile Intervention Kit. The reviewer, FM, and handling editor declared their shared affiliation and the handling editor states that the process nevertheless met the standards of a fair and objective review.

## Footnotes

^1^Aronson ID, Guarino H, Bennett AS, Marsch LA, Gwadz M, Cleland CM, et al. (2016) (submitted).
